# *Haemophilus influenzae* blood-stream infection and third-generation cephalosporin susceptibility testing: a comparative case study using EUCAST and CLSI guidelines

**DOI:** 10.1099/acmi.0.000578.v4

**Published:** 2023-10-09

**Authors:** John Merlino, Sophia Rizzo, Suzanne English, Sai Rupa Baskar, Steven Siarakas, Genevieve Mckew, Shelanah Fernanado, Timothy Gray

**Affiliations:** ^1^​ Department of Microbiology and Infectious Diseases, Concord Hospital, NSW Health Pathology, NSW Health, Concord, Australia; ^2^​ School of Medical Sciences, Department of Infection and Immunity, Faculty of Medicine and Health, University of Sydney, Camperdown, Australia; ^3^​ Concord Hospital Clinical School, Faculty of Medicine and Health, University of Sydney, Camperdown, Australia

**Keywords:** BLNAR, cefotaxime, ceftriaxone, CLSI, EUCAST, *Haemophilus influenzae*, resistance, susceptibility

## Abstract

**Introduction.:**

In this comparative case study, we discuss clinically relevant discrepancies of antimicrobial susceptibility testing (AST) interpretation for ceftriaxone against a non-typable, beta-lactamase negative, ampicillin-resistant (BLNAR) *Haemophilus influenzae* isolated from a blood culture.

**Case report.:**

A 74-year-old man presented with a 3 day illness characterized by shortness of breath and dry cough, and was noted to be febrile and hypoxic on admission. A blood culture bottle flagged positive with Gram-negative coccobacilli, later identified as *Haemophilus influenzae* with the patient commenced on ceftriaxone. The isolate was beta-lactamase negative and antibiotic susceptibility testing (AST) using disc diffusion revealed the isolate resistant to ceftriaxone and ampicillin by EUCAST methodology, with the patient subsequently changed to amoxicillin/clavulanate. Further AST using the CLSI methodology in parallel demonstrated discrepant results between the two susceptibility methods. The patient recovered without complications.

**Conclusion.:**

This discrepancy could lead to inconsistent reporting of susceptibilities between laboratories, and consequently antibiotic prescribing, especially for invasive isolates. As more laboratories adopt EUCAST methodologies for AST interpretation in Australia and globally, it is important for clinicians to consider the clinical implications of these methodological discrepancies.

## Data Summary

No data was generated during this research or is required for the work to be reproduced.

## Introduction

*Haemophilus influenzae* (HI) is a small fastidious Gram-negative bacterium, associated with a spectrum of infections, ranging from mild infections, such as otitis media or bronchitis, to life-threatening infections, such invasive disease or epiglottitis. While HI serotype B is now rare in Australia due to mass vaccination, non-B HI invasive disease, namely blood-stream infections and meningitis, continue to cause significant morbidity and risk of mortality [1]. Antimicrobial susceptibility testing (AST) of HI isolates from life-threatening infections remains essential to targeting therapy at the individual level as well as empirical therapy prescribing through population-level surveillance [2, 3]. Third-generation cephalosporins, such as ceftriaxone and cefotaxime, are broad spectrum antibiotics commonly used in Australia, and remain critical components of life-saving empirical therapy for suspected meningitis, as well as a range of severe Gram-negative infections, including blood-stream infections [1].

Therefore, discrepancies in susceptibility reporting for third-generation cephalosporins between the two of the most common AST methodologies [4, 5] used by laboratories have major implications for population-level empirical treatment of life-threatening infections, as well as individual patient care. We have previously demonstrated how EUCAST Version 12 and CLSI methodologies give clinically relevant, discrepant results for classifying isolates of HI as beta-lactamase negative, ampicillin-resistant (BLNAR) [6]; although BLNAR detection has major implications for aminopenicillin and amoxicillin-clavulanic acid prescribing, it is increasingly recognized to be a signal for risk of resistance to third-generation cephalosporins in Europe and Japan via acquisition of mutations within penicillin-binding-protein-3 (PBP3) [7–13]. Nevertheless, implementing the EUCAST methodology means that a clinician would be more likely to change antibiotics to a more restricted agent (e.g. moxifloxacin), with implications for antimicrobial stewardship [14, 15].

However, PBP3 target alteration is rarely mediated by single mutations, and consistent correlation between PBP3 genotype and resistance phenotype has proven difficult, which can be explained at least in part by considerable heterogeneity of sequence types (STs) between isolates of non-B serotype HI [8]. This genotypic/phenotypic uncertainty, combined with a paucity of published clinical outcome data comparing the EUCAST (version 12) and CLSI (M100-S31, 2021) methodologies, creates a dilemma with respect to treatment decisions and antimicrobial stewardship of restricted antibiotics in the Australian context. In this comparative case study, we demonstrate the critical differences in laboratory methodologies and interpretations may have a significant impact on management of life-threatening infection with BLNAR HI, with wider implications for empirical antibiotic choice.

## Case report

We describe the case of a 74-year-old man who presented to the Emergency Department with a 3 day illness characterized by shortness of breath and dry cough. His past medical history included obstructive sleep apnoea, chronic obstructive airway disease, non-insulin-dependent diabetes, hypertension and hypercholesterolemia. On admission, he was noted to be febrile (38.2 °C), tachypnoeic (28 breaths/min), hypoxic (requiring 4 l of oxygen via nasal prongs to maintain peripheral oxygen saturation >95 %), tachycardic (133 beats/min) with an irregular heart rhythm, and he was also noted to have left-sided chest crepitations. There was no clinical evidence of delirium or meningoencephalitis.

Initial blood investigations revealed an elevated peripheral white blood cell count (27.3×10^9^/l) with neutrophilia (25.4×10^9^/l), and an elevated C-reactive protein (163 mg l^−1^). Chest x-ray findings were consistent with multi-lobar consolidation and he returned a negative nasopharyngeal/throat swab for SARS-CoV-2. He was admitted to hospital with a diagnosis of severe community-acquired pneumonia (SMART-COP=5) and commenced intravenous antibiotic treatment with ceftriaxone (2 g daily) and azithromycin (500 mg daily) [1].

An aerobic blood culture bottle collected prior to antibiotics and incubated on the automated BacT/Alert blood culture system (bioMerieux, Australia) flagged positive on day 1 of admission, with Gram negative coccobacilli on Gram stain, and was subcultured to routine media. An isolate grown on chocolate agar after 4 h was identified by MALDI-TOF mass spectrometry (Bruker, Australia) as *Haemophilus influenzae* (HI, score=2.14). In light of this result, azithromycin was ceased after two doses and ceftriaxone 2 g daily was continued empirically as monotherapy for invasive HI, pending the results of AST. The patient’s clinical course improved, with resolution of his fevers and reduction in his inflammatory markers.

Subsequently, the HI isolate was reported as beta-lactamase negative, but resistant to ceftriaxone and ampicillin (EUCAST version 12.0, 2022, methodology) [4], so monotherapy with ceftriaxone was changed on day 4 of admission to intravenous amoxicillin/clavulanate (1000/200 mg 8-hourly); on day 6 of admission, the patient was changed to oral amoxicillin/clavulanate (875/125 mg 12-hourly) to complete a further 5 days of therapy. He was discharged home on day 10 of admission without any complications. The patient’s isolate was later sent to a reference laboratory for molecular serotyping, where it was reported as non-typable HI.

In light of the unusual and clinically important result for ceftriaxone on an invasive isolate, the AST process for this specimen was reviewed in detail. Beta lactamase testing was negative by Nitrocefin disc test (Thermofisher, Australia). AST testing was performed using disc diffusion testing using commercial solid media and antibiotic impregnated discs (Thermofisher, Australia). MIC values were determined by gradient diffusion on solid media with commercial epsilometer (E)-test strips (BioMerieux, Australia). For AST by the EUCAST (version 12.0, 2022) disc and E-test methodologies, the isolate was inoculated on Mueller–Hinton agar with 5 % defibrinated horse blood and 20 mg l^−1^ NAD (Thermofisher, Australia), and incubated in 5 % CO_2_ at 35–37 °C for 18–20 h.

We retested the isolate by the EUCAST (version 12.0, 2022) method [4] and performed parallel testing using the CLSI (M100-S31, 2021) methodology [5] as a comparator, inoculating the isolate on HTM media and incubated in 5 % CO_2_ at 35–37 °C for 16–18 h. Both EUCAST (version 12.0, 2022) and CLSI (M100-S31, 2021) methods required discs containing ceftriaxone 30 ug, while only EUCAST had breakpoints for cefotaxime (5 ug disc) [4]. The EUCAST (version 12.0, 2022) flow chart [4] based on the penicillin 1 µg disc screen test for beta-lactam resistance mechanism was followed and found to be resistant and further susceptibility tests performed for the relevant agents and interpreted according to their breakpoints (Table 1). Inhibition disc zone diameters (DD) were measured using callipers. For quality control, *Haemophilus influenzae* ATCC 49247 was used. Our results, shown in Table 1, demonstrate discrepant third-generation cephalosporin AST interpretations despite identical values obtained for disc zone diameters and MICs by the different methodologies, and indicate that differences in media may not be significant for testing these antibiotics against HI.

**Table 1.** E-test MIC values, disc zone diameters and interpretations of antibiotics for the *Haemophilus influenzae* strain detected in blood culture using third-generation cephalosporins

		MIC (mg l^–1^)				Disc zone diameter (mm)			
		AMP	PEN	CRO	CTX	AMP 2 µg	PEN 1 µg	CRO 30 µg	CTX 5 µg
Breakpoints	EUCAST	1	n/a	0.125	0.125	18	12	32	27
	CLSI	n/a	n/a	<=2	<=2	n/a	n/a	26	n/a
Patient results	EUCAST	32	–	0.5 (R)	0.75 (R)	11 (R)	6 (R)	30 (R)	17 (R)
	CLSI	–	–	0.5 (S)	0.75 (S)	–	–	30 (S)	–

CRO ceftriaxone, CTX cefotaxime. AMP ampicillin, PEN penicillin.

AST, Animicrobial Susceptibility testing.

## Discussion

There has been little specific information published on clinical outcomes of invasive infections caused by HI strains demonstrating discrepant ceftriaxone- or cefotaxime-resistant interpretations between EUCAST (version 12.0, 2022) and CLSI (M100-S31, 2021) methodologies. Furthermore, there is limited published information on outcomes for invasive infections caused by strains that are demonstrated to have MICs of 0.125 and 2 mg l^−1^ by either methodology.

Our clinical case demonstrates how MIC breakpoints for third-generation cephalosporins differ between the EUCAST (version 12.0, 2022) and CLSI (M100-S31, 2021) methodologies for invasive infections caused by HI. This is a major concern for clinical laboratories, especially for those located in regions where invasive beta-lactamase negative, ampicillin-resistant (BLNAR) isolates of HI are very common. EUCAST (version 12.0, 2022) MIC breakpoints values for cefotaxime and ceftriaxone are 0.125 mg l^−1^, while CLSI (M100-S31, 2021) MIC breakpoints levels are 2 ug ml^−1^; Nurnberg and colleagues describe in detail that this difference is due to different methodological approaches in breakpoint determination for HI, including the application of underlying assumptions in PK/PD modelling [16]. In addition, Nurnberg and colleagues also pointed out in their study that this reduced susceptibility to third-generation cephalosporins is not as rare in BLNAR isolates of HI as suggested by EUCAST (version 12.0, 2022) recommendations [16].

While whole-genome sequencing (WGS) is not routinely available in clinical laboratories to guide decision making, it has provided some mechanistic insights into the clinical relevance of these discrepant breakpoint interpretations. WGS of isolates submitted for population-level surveillance is increasingly informing a conservative approach to breakpoint selection, as genetic mechanisms of selection are identified that produce higher MIC values *in vitro*. For example, the high-level resistance from the expression of TEM-1 beta-lactamase genes has informed routine laboratory testing of HI for decades, usually rapidly detectable through Nitrocefin disc test [2]. However, WGS has shown that HI may harbour non-synonymous mutations in the *ftsI* gene, producing amino acid substitutions in penicillin-binding protein 3 (PBP3), which may confer *in vitro* resistance to cefotaxime [7–9, 11]. Such resistance is more problematic to detect without conservative breakpoints, but even conservative breakpoints can fail to distinguish non-wild-type isolates [7, 11]. In addition, at least one French study has suggested that the genotype and phenotype of invasive and non-invasive strains carrying *ftsI* gene mutations could be sufficiently different, such that the detection of these mutations in an invasive isolate may be insufficient to predict clinical outcome [8].

Furthermore published evidence on the correlation between PBP3 mutations, *in vitro* MICs of 0.25–2 mg l^−1^, and clinical outcome remains scarce and contradictory. Skaare *et al*. reported a Norwegiuan HI from a blood-stream infection in an immunocompromised patient, with an MIC ≤0.12 mg l^−1^ (susceptible; EUCAST version 12.0, 2022, methodology); the patient responded well to an initial 3 days of cefotaxime before being changed to oral ciprofloxacin, but the isolate was subsequently found to have the *ftsI* mutations, resulting in PBP3 amino acid substitutions (S385T and N526K) [11]. Thomas *et al*. reported a non-invasive French BLNAR isolate from the respiratory tract of an immunocompromised patient with chronic lung disease; the cefotaxime MIC was 0.25 mg l^−1^ (resistant; EUCAST version 12.0, 2022, methodology) and ceftriaxone MIC of 0.094 mg l^−1^ (susceptible; EUCAST version 12.0, 2022, methodology) and both clinical and microbiological failure was reported after 8 days of IV ceftriaxone [7]. The isolate was subsequently found to have the *ftsI* mutations, producing PBP3 amino acid substitutions (S357N, M377I, S385T, L389F, A502T and N526K). Nishimura *et al*. reported a Japanese non-typeable BLNAR HI causing bacteraemia, secondary to infection of a massive uterine adenomyoma with endometritis; the isolate returned an MIC of 1 mg l^−1^ to cefotaxime (susceptible; CLSI M100-S31, 2021 methodology), and the patient became afebrile after 7 days of ceftriaxone, despite source control not occurring for a further 10 days (*ftsI* sequencing was not performed) [13].

## Conclusion

We have demonstrated that two of the most common AST methodologies used in Australian and other global laboratories, when applied to an invasive non-typeable BLNAR HI isolate, produce discrepant results for the most commonly prescribed antibiotics used empirically for this life-threatening infection. Given these discrepancies, and the lack of available clinical outcome for isolates with discrepant results, we cannot say whether or not the patient had a favourable outcome due to the presence of azithromycin in the empirical therapy and its long half-life after cessation, or whether the ceftriaxone therapy was itself sufficient. However, this case demonstrates that the clinical decision to change to intravenous amoxycillin/clavulanate (1000/200 mg 8-hourly) was clearly based on the AST results achieved with the EUCAST methodology, and it remains unclear if the patient would have relapsed had the conflicting results of the CLSI methodology been used instead.

Therefore, clinicians should be aware of this possibility when encountering life-threatening infections caused by non-typeable, BLNAR HI isolates. Clearly, further research is required to determine the clinical implications of these methodological discrepancies for individual patient care and antimicrobial stewardship, as well as population-level determinations of empirical antibiotic regimens.
